# Predictors of High School Students’ Intentions and Behaviors in Using AI Learning Tools: An Extended Theory of Planned Behavior Approach

**DOI:** 10.3390/bs16050736

**Published:** 2026-05-09

**Authors:** Ziqi Zhang, Fuhai An

**Affiliations:** 1 Jing Hengyi School of Education, Hangzhou Normal University, Hangzhou 311121, China; zhangziqi77@stu.hznu.edu.cn; 2 Chinese Education Modernization Research Institute, Jing Hengyi School of Education, Hangzhou Normal University, Hangzhou 311121, China

**Keywords:** AI learning tools, theory of planned behavior, AI anxiety, intention, learning behavior, high school students

## Abstract

AI learning tools are now common enough in secondary education that students are expected to know how to use them, yet we still know relatively little about what drives their use among high school students. This study applied an extended theory of planned behavior (TPB) model to examine whether affective attitude, instrumental attitude, subjective norms, perceived behavioral control, and AI anxiety predicted students’ intention to use AI learning tools and their use behaviors, the latter encompassing three dimensions: seeking AI help, evaluating AI responses, and applying AI output. Attitude was divided into affective and instrumental components, and AI anxiety was added as a separate emotional predictor. Data were collected from 513 students in three public high schools in Hangzhou, China, and analyzed with structural equation modeling. Affective attitude, instrumental attitude, subjective norms, and perceived behavioral control all positively predicted intention. AI anxiety did not significantly predict intention, but it negatively predicted seeking AI help, evaluating AI responses, and applying AI output. The results suggest that, for these students, AI anxiety is more closely associated with behavioral engagement than intention formation. This distinction helps to clarify how adolescents use AI learning tools and may inform more focused school support for responsible AI use.

## 1. Introduction

Generative artificial intelligence and related AI learning tools are reshaping how students access information, complete academic tasks, and receive feedback. Large language models, intelligent tutoring systems, and AI-driven learning platforms can provide explanations, examples, practice recommendations, and writing feedback, thereby supporting students’ learning processes to a certain extent (Chiu, 2024; Crompton & Burke, 2023; Kasneci et al., 2023; Lim et al., 2023). In the context of Chinese K-12 education, domestic AI applications such as Doubao, ERNIE Bot, and iFlytek Spark have gradually entered classroom instruction, after-school learning, and self-directed learning scenarios, while relevant policies continue to emphasize AI literacy education and the integration of intelligent technologies with education (Chai et al., 2020; Ng et al., 2021; Ng et al., 2023b; Sing et al., 2022). However, the increasing prevalence of AI in schools has also raised concerns about potential risks, such as over-reliance on AI-generated content, diminished independent thinking, and academic integrity issues (Chan & Hu, 2023; Cotton et al., 2024). At the same time, the growing availability of AI does not necessarily mean that students will use these tools consistently, appropriately, or effectively (Strzelecki, 2024). Therefore, understanding the psychological and social factors associated with students’ use of AI learning tools has become an important topic in educational research and practice.

Prior research has provided a useful starting point, but two limitations remain. First, much of the existing work on educational technology adoption has focused on general acceptance or behavioral intention, with less attention to high school students, who operate within a more tightly regulated learning environment (Ng et al., 2023a; Scherer et al., 2019). Compared with university students, high school students are more likely to be influenced by teachers’ expectations, parental monitoring, time constraints, and school rules. Their intentions and behaviors related to AI use may therefore follow a somewhat different logic. Second, existing studies have often treated AI use as a single outcome, with insufficient attention to different stages of students’ interaction with AI (Lim et al., 2023). In practice, students’ willingness to seek help from AI, critically evaluate AI-generated responses, and apply AI output to learning tasks represent qualitatively different forms of engagement. Treating them as a single undifferentiated construct risks obscuring the distinct psychological mechanisms that drive each type of behavior (Ouyang & Jiao, 2021).

The theory of planned behavior (TPB) provides a useful starting point for this question (Scherer & Teo, 2019). In the formulation of Ajzen (1991), behavioral intention is jointly determined by three conceptually independent antecedents (attitude toward the behavior, subjective norms, and perceived behavioral control) and intention, in conjunction with perceived behavioral control, then predicts the actual performance of the behavior. TPB has been used widely in studies of educational behavior and technology adoption, including work on AI learning, self-assessment, and learning engagement (Armitage & Conner, 2001; Chai et al., 2020). Even so, the standard TPB variables may not capture the full range of students’ responses to AI-related tools. Ajzen (1991) explicitly stated that the standard set of TPB predictors is not exhaustive: additional variables may be incorporated, but only when two conditions are jointly met. First, the candidate variable must demonstrate incremental validity; that is, it should explain variance in intention or behavior over and above attitude, subjective norms, and perceived behavioral control (Armitage & Conner, 2001; Chai et al., 2020). Second, its inclusion must be theoretically justified, in the sense that the proposed variable captures a conceptually distinct mechanism rather than a redundant indicator of an existing TPB construct (Rivis et al., 2009). Following this logic, AI anxiety satisfies both conditions in the present context. Conceptually, it captures a domain-specific affective response to AI technology that is qualitatively different from a generic evaluative attitude or perceived control judgment. Empirically, prior work has shown that AI-related anxiety predicts technology-use behavior beyond the variance accounted for by classical TPB or TAM constructs (Budhathoki et al., 2024; Y.-M. Wang et al., 2022). Including AI anxiety in the present model is therefore consistent with Ajzen’s (1991) original criteria for legitimate TPB extensions, rather than an ad hoc addition. Researchers have therefore examined variables such as moral norms and anticipated affect as possible extensions (Rivis et al., 2009). That move makes sense in the present context. AI learning tools bring with them concerns about misinformation, overreliance, academic integrity, and even technological replacement. Those concerns may take the form of AI anxiety, which could help to explain how students approach these tools (Grassini, 2023; Y.-Y. Wang & Wang, 2022).

Based on this rationale, the present study extends the TPB framework by incorporating AI anxiety and by recognizing that using AI learning tools is itself a behavior that can be decomposed into three distinct components—namely, seeking AI help, evaluating AI responses, and applying AI output—each serving as a separate outcome variable. This treatment is grounded in two considerations. First, students’ interactions with AI are not single acts but continuous processes that involve obtaining information, judging information, and using information. Second, different stages of this process may be predicted by different factors; for example, students may report a strong intention to use AI while still being reluctant to adopt AI output in real academic tasks. Likewise, they may be willing to ask AI for help without carefully evaluating the quality of its responses. Distinguishing among these dimensions allows a more precise account of the behavioral structure underlying students’ use of AI learning tools.

To this end, the present study used data from 513 Chinese high school students to address two research questions:

(a) Which factors predict high school students’ intention to use AI learning tools?
(b) Which factors predict students’ self-reported behaviors in seeking AI help, evaluating AI responses, and applying AI output?

This study makes three main contributions: it incorporates AI anxiety as a supplementary affective predictor into the TPB framework and reveals that its association with self-reported behavioral engagement follows a path distinct from that of the core TPB constructs; it operationalizes AI learning tool use as a multidimensional behavioral construct encompassing seeking, evaluating, and applying, thereby providing a more fine-grained picture of how students interact with AI; and it offers empirical evidence from an understudied population of Chinese high school students, contributing to a broader understanding of AI-assisted learning at the secondary education level.

## 2. Literature Review

### 2.1. The Theory of Planned Behavior and Its Application in Educational Technology Research

According to the theory of planned behavior, the proximal determinants of whether an individual performs a given behavior are behavioral intention and perceived behavioral control. Intention, in turn, is shaped by three conceptually independent antecedents: attitude, defined as an individual’s positive or negative evaluation of performing the behavior; subjective norms, reflecting perceived expectations from important referent groups (e.g., teachers, parents, and peers) regarding whether the individual should perform the behavior; and perceived behavioral control, representing an individual’s confidence in possessing the skills, resources, and opportunities needed to successfully execute the behavior.

TPB has demonstrated substantial applicability in research on educational technology and learning-related behavior. Chai et al. (2020) found that secondary school students’ intentions to learn AI were significantly predicted by attitude, subjective norms, and perceived behavioral control. Yan et al. (2020b) similarly validated TPB pathways in the context of student self-assessment. In research on teachers’ technology adoption, Teo et al. (2016) further reported that attitude and perceived behavioral control were important predictors of intention to use technology. Together, these findings suggest that TPB is suitable not only for general technology adoption but also for self-regulated and learning-related behaviors in educational contexts. More recently, Strzelecki (2024) applied an extended UTAUT model to examine ChatGPT acceptance among university students, further confirming the relevance of attitude and social influence in AI adoption decisions.

### 2.2. AI Learning Tool Use Behavior

Using AI learning tools is a behavior. Although this behavior is often treated as a single outcome in prior research, the learning process suggests that it encompasses at least three related yet distinguishable stages. First, students must decide whether to seek help from AI tools, such as by requesting explanations, examples, or feedback. Second, they need to determine whether AI-generated content is trustworthy and appropriate for their learning needs. Third, they need to integrate AI-provided information into their actual learning activities. If these stages are collapsed into one undifferentiated construct, important behavioral distinctions may be obscured, and the differential effects of various predictors on each stage cannot be examined.

The present study drew on the cyclical self-assessment process model proposed by Yan and Brown (2017) and on feedback process theory articulated by Hattie and Timperley (2007) to conceptualize AI learning tool use as comprising three dimensions: seeking AI help, evaluating AI responses, and applying AI output. Although interacting with AI is not identical to receiving feedback from teachers or peers, both involve information acquisition, critical judgment, and subsequent use. Recent research on AI in education has likewise described learners’ interactions with AI as combining information seeking and feedback use (Chiu et al., 2023; Ouyang & Jiao, 2021).

Seeking AI help (SA) refers to students’ active attempts to obtain learning support from AI tools, such as asking for explanations, examples, or suggestions. Evaluating AI responses (EA) refers to students’ critical processing of AI-generated content, including comparison, verification, and judgments about usefulness. Applying AI output (AA) refers to students’ reported use of AI-generated information to revise work, adjust strategies, or solve learning problems. These dimensions are distinguished because students may be active in one stage while remaining cautious in another. Asking AI a question is a relatively low-threshold initiating behavior, whereas incorporating AI output into one’s own academic work typically involves greater responsibility and judgment (Hattie & Timperley, 2007). A multidimensional conceptualization therefore provides a more refined account of the internal structure of students’ AI learning tool use.

### 2.3. Predictors of AI Learning Tool Use Behavior

Within the TPB framework, the present study identified four key predictors of students’ intention to use AI learning tools: affective attitude, instrumental attitude, subjective norms, and perceived behavioral control. Following Ajzen (2002), attitude toward a behavior can be decomposed into two conceptually distinct dimensions. The affective component reflects the emotional experience associated with performing the behavior (e.g., enjoyment, interest), whereas the instrumental component reflects cognitive evaluations of the behavior’s outcomes (e.g., usefulness, value). This distinction is well established in TPB research and allows for a more precise examination of how different facets of attitude contribute to intention formation. In the context of AI learning tools, affective attitude captures whether students find the experience of using these tools pleasant and engaging, while instrumental attitude captures whether students perceive these tools as useful and beneficial for their learning outcomes.

Subjective norms refer to the social pressure students perceive from people who matter to them (Ajzen, 1991). In school settings, those people usually include teachers, parents, and peers. Prior studies have shown that subjective norms matter for technology adoption in education. Chai et al. (2020) found that subjective norms significantly predicted Chinese secondary school students’ intentions to learn AI. Sing et al. (2022) also reported positive links between subjective norms and students’ intentions to learn AI, with readiness and social good acting as moderators. This influence may be especially strong among high school students, who study in settings where teacher approval, peer practice, and parental expectations are hard to ignore.

Perceived behavioral control reflects individuals’ confidence in their ability to perform the target behavior, encompassing judgments about the availability of necessary skills, resources, and opportunities. In the TPB framework, perceived behavioral control is unique in that it predicts both intention and behavior directly. Students who believe they possess the technical competence and access needed to use AI learning tools effectively are more likely to form positive intentions and to translate those intentions into action. In the context of AI education, Chai et al. (2020) found that perceived behavioral control was a significant predictor of secondary school students’ intentions to learn AI, and Teo et al. (2016) similarly reported that perceived behavioral control was an important predictor of teachers’ intention to use technology. These findings suggest that perceived behavioral control is a robust predictor across different educational technology adoption contexts.

### 2.4. The Potential Role of AI Anxiety in an Extended TPB Framework

(Ajzen, 1991) noted that TPB can be extended when additional variables explain variance beyond the original constructs. Researchers have therefore considered variables such as moral norms and anticipated affect (Rivis et al., 2009). AI learning tools are a good case for such an extension because students’ responses to them are shaped not only by usefulness or social expectations, but also by worries that fall outside the standard TPB variables.

AI anxiety refers to feelings of worry, unease, or tension when individuals encounter AI technologies (Y.-Y. Wang & Wang, 2022). Such emotional reactions may stem from unfamiliarity with technical complexity, concerns about inaccurate information, worries about academic integrity, or fears that AI may replace human capabilities (Kasneci et al., 2023).

AI anxiety may also work differently from the core TPB variables when intention and behavior are separated. Attitude, subjective norms, and perceived behavioral control are tied closely to students’ evaluations of a target behavior, so they should feed more directly into intention. AI anxiety is broader and less deliberative. Students may still report that they intend to use AI, yet hesitate when they actually need to ask for help, judge an answer, or use AI output in schoolwork (Venkatesh et al., 2003).

This issue is particularly salient in secondary education, where high school students may hold positive evaluations of AI yet worry about the risks associated with inappropriate use under school discipline and examination norms. Venkatesh et al. (2003) noted that anxiety-related variables tend to be more closely associated with behavioral barriers than with the core cognitive judgments involved in intention formation, and recent studies have confirmed this pattern in AI contexts; for example, Y.-M. Wang et al. (2022) demonstrated that AI anxiety influenced students’ learning behavior primarily through indirect pathways rather than directly shaping intention, while Budhathoki et al. (2024) reported that anxiety exhibited different predictive patterns for intention versus actual adoption behavior.

A central question for the present model is whether AI anxiety should be specified as influencing behavior directly, indirectly through intention, or along both pathways simultaneously. Three converging lines of theoretical and empirical reasoning support the inclusion of a direct path that is not fully absorbed by intention. First, dual-process accounts of technology use distinguish between deliberative cognitive appraisals, such as usefulness, social legitimacy, and perceived control, which primarily drive the formation of intention, and more automatic affective reactions, which tend to surface at the moment of behavioral enactment according to Venkatesh et al. (2003). Anxiety, as a relatively automatic and aversive emotional response, is more likely to operate through this second route than through deliberative appraisal. Second, within UTAUT and its extensions, anxiety has been repeatedly modeled and observed as a determinant of usage behavior rather than solely of intention (Venkatesh, 2000), and recent AI-specific evidence indicates that AI anxiety constrains adoption behaviors even when intention remains positive, according to Budhathoki et al. (2024). Third, in the high school context examined here, the gap between expressing an intention to use AI and actually deploying it in a graded task is theoretically meaningful, because students may rationally endorse AI use yet still hesitate at the point of execution because of concerns about misinformation, overreliance, or academic-integrity scrutiny, according to Venkatesh et al. (2003). Taken together, these considerations indicate that AI anxiety can plausibly affect AI learning tool use through two non-redundant routes: an indirect route operating via intention, in line with the standard TPB structure, and a direct route operating at the point of behavioral enactment, in line with the dual-process account outlined above. The present model therefore specifies both pathways simultaneously, which permits each route to be estimated and reported on its own terms.

Including AI anxiety as an extended variable in the TPB framework is therefore empirically justified and theoretically useful for examining whether the influence of emotional factors on AI learning tool use differs from that of classical cognitive predictors.

### 2.5. The Present Study and Hypotheses

Based on this review, the study proposed an extended TPB model in which affective attitude, instrumental attitude, subjective norms, perceived behavioral control, and AI anxiety were treated as antecedents; intention was modeled as a mediator; and AI learning tool use was divided into three outcomes: seeking AI help, evaluating AI responses, and applying AI output. Following TPB, affective attitude, instrumental attitude, subjective norms, and perceived behavioral control were expected to predict intention positively. AI anxiety was expected to relate negatively to intention and to the three behavioral dimensions. Intention, perceived behavioral control, and AI anxiety were also expected to predict the three behavioral dimensions directly. The hypotheses are listed below.

Predictors of intention (RQ1):

**H1a.**  *Affective attitude (AAT) positively predicts intention to use AI learning tools.*

**H1b.**  *Instrumental attitude (IAT) positively predicts intention to use AI learning tools.*

**H1c.**  *Subjective norms (SNS) positively predict intention to use AI learning tools.*

**H1d.**  *Perceived behavioral control (PBC) positively predicts intention to use AI learning tools.*

**H1e.**  *AI anxiety (AIA) negatively predicts intention to use AI learning tools.*

Predictors of behavior (RQ2):

**H2a.**  *Intention (INT) positively predicts seeking AI help, evaluating AI responses, and applying AI output.*

**H2b.**  *Perceived behavioral control (PBC) positively predicts seeking AI help, evaluating AI responses, and applying AI output.*

**H2c.**  *AI anxiety (AIA) negatively predicts seeking AI help, evaluating AI responses, and applying AI output.*

**Figure 1:**
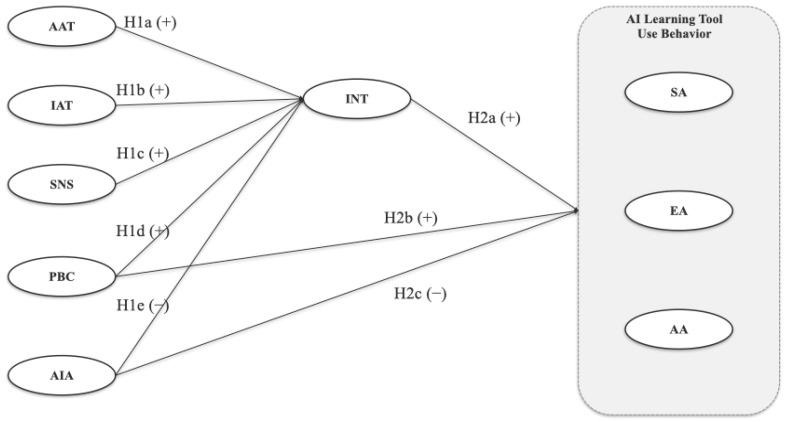
Hypothesized model.

Therefore, the theoretical research model of this study is as follows. Figure 1 presents the conceptual model depicting these relationships.

## 3. Method

### 3.1. Participants and Procedures

The participants were students from three public high schools in Hangzhou, China. A stratified sampling approach was used to recruit students from Grades 10 and 11 across the participating schools to ensure a roughly balanced grade distribution. The study excluded Grade 12 students because their curriculum was almost entirely devoted to examination review, leaving minimal opportunity for exploratory use of AI learning tools. All participating schools had already introduced domestic AI learning tools, such as Doubao, ERNIE Bot, and SparkDesk, into instructional or after-school learning activities. As a result, the surveyed students had some degree of experience with AI-supported learning. This sampling strategy helped to ensure that participants could meaningfully respond to the questionnaire, although it also means that the findings are most applicable to students who already have some exposure to AI learning tools.

A total of 550 questionnaires were distributed. After excluding incomplete responses, clearly patterned responses (e.g., invariant or highly repetitive responding across long item sequences), and responses with obvious internal inconsistencies, 513 valid questionnaires were retained, yielding an effective response rate of 93.3%. All 513 questionnaires retained for analysis were complete with no missing data. Table 1 presents the descriptive statistics of the sample. In the Chinese education system, grade level is closely linked to age due to the structured academic progression system; therefore, grade level was retained as a demographic variable rather than age.

Participants were between 15 and 17 years old. Data collection took place over a 2-week period during a regular school semester. After school permission had been obtained, the research team organized students to complete an online questionnaire via the Wenjuanxing platform in school computer rooms. The questionnaire was administered in organized school sessions, and the average completion time was approximately 15 to 20 min. To reduce the potential influence of common method bias, several procedural remedies were used, including emphasizing anonymity and confidentiality on the first page of the questionnaire, separating predictor and outcome measures across different pages, and varying response directions across measures when appropriate. All valid respondents received school supplies as compensation. The study procedures were approved by the ethics review process of the researchers’ institution and the participating schools. All participants and their guardians were informed of the study purpose and procedure before data collection, and written informed consent was obtained.

### 3.2. Measures

All constructs in this study were measured with established scales that were adapted to the context of high school students’ use of AI learning tools. All items were presented in simplified Chinese and were rated on a 5-point Likert scale ranging from 1 (strongly disagree) to 5 (strongly agree). Item development and adaptation were guided by the recommendations of Ajzen (2002) for TPB questionnaire construction.

To ensure cross-cultural appropriateness and content validity, a systematic adaptation procedure was employed. In the first stage, two bilingual researchers with backgrounds in educational psychology independently produced Chinese translations of all items. The two versions were then compared, and discrepancies were resolved through joint discussion until consensus was reached. In the second stage, an independent translator with no prior exposure to the original instruments performed a back-translation into English. The research team evaluated the back-translated version against the source items and refined the wording where necessary to preserve semantic equivalence. In the third stage, an expert panel was convened to review the adapted items. The panel comprised three academics specializing in psychometrics and educational measurement, and three practicing high school teachers with experience in AI-integrated instruction. Panelists evaluated each item for linguistic clarity, developmental appropriateness for high school students, and alignment with the intended construct. Minor revisions were made on the basis of their feedback. The final adapted questionnaire items are presented in the Supplementary Materials (Table S1).

#### 3.2.1. Affective Attitude (AAT)

The affective attitude scale included four items developed in accordance with Ajzen (2002) TPB questionnaire guidelines. It measured students’ emotional responses to using AI learning tools. A sample item was “Using AI learning tools is enjoyable.” Cronbach’s alpha was 0.89. The single-factor CFA indices were χ^2^/df = 3.722, CFI = 0.995, TLI = 0.986, RMSEA = 0.073, and SRMR = 0.013.

#### 3.2.2. Instrumental Attitude (IAT)

The instrumental attitude scale included four items adapted from Venkatesh et al. (2003) perceived usefulness scale and Chai et al. (2020) AI learning attitude scale. It assessed students’ evaluations of the value and outcomes of using AI learning tools. A sample item was “Using AI learning tools helps me understand my strengths and weaknesses in learning.” Cronbach’s alpha was 0.85. The single-factor CFA indices were χ^2^/df = 3.308, CFI = 0.995, TLI = 0.984, RMSEA = 0.067, and SRMR = 0.015.

#### 3.2.3. Subjective Norms (SNS)

The subjective norms scale included four items constructed based on Ajzen (2002) TPB questionnaire guidelines. It measured students’ perceptions of important others’ expectations regarding their use of AI learning tools. A sample item was “My teachers think I should use AI learning tools to support my learning.” Cronbach’s alpha was 0.81. The single-factor CFA indices were χ^2^/df = 3.528, CFI = 0.992, TLI = 0.977, RMSEA = 0.070, and SRMR = 0.019.

#### 3.2.4. Perceived Behavioral Control (PBC)

The perceived behavioral control scale included six items adapted from Yan et al. (2020a) self-assessment PBC scale and Ajzen’s (2002) TPB questionnaire guidelines. It assessed students’ judgments about their own ability, resources, and sense of control regarding the use of AI learning tools. A sample item was “I have sufficient knowledge to use AI learning tools effectively.” Cronbach’s alpha was 0.90. The single-factor CFA indices were χ^2^/df = 1.714, CFI = 0.996, TLI = 0.993, RMSEA = 0.037, and SRMR = 0.016.

#### 3.2.5. AI Anxiety (AIA)

The AI anxiety scale included five items selected from the learning anxiety subscale of the artificial intelligence anxiety scale developed by (Y.-Y. Wang & Wang, 2022). It measured students’ worries and uneasiness when using AI technologies in learning contexts. A sample item was “I feel anxious when I need to use AI tools for learning tasks.” Cronbach’s alpha was 0.87. The single-factor CFA indices were χ^2^/df = 1.936, CFI = 0.996, TLI = 0.992, RMSEA = 0.043, and SRMR = 0.015.

#### 3.2.6. Intention (INT)

The intention scale included four items adapted from (Chai et al., 2020) AI learning intention scale and Ajzen’s (2002) TPB questionnaire guidelines. It measured students’ willingness to continue using AI learning tools in the future. A sample item was “I plan to use AI learning tools to support my learning in the future.” Cronbach’s alpha was 0.84. The single-factor CFA indices were χ^2^/df = 3.024, CFI = 0.995, TLI = 0.985, RMSEA = 0.063, and SRMR = 0.016.

#### 3.2.7. Seeking AI Help (SA)

The seeking AI help scale included four items adapted from Yan’s (2018) Self-Assessment Practice Scale (SaPS), with the original self-assessment context modified to reflect AI learning tool use. It measured students’ active efforts to obtain learning support from AI tools. A sample item was “When I have learning questions, I seek help from AI tools.” Cronbach’s alpha was 0.86. The single-factor CFA indices were χ^2^/df = 5.468, CFI = 0.990, TLI = 0.971, RMSEA = 0.093, and SRMR = 0.018.

#### 3.2.8. Evaluating AI Responses (EA)

The evaluating AI responses scale included four items adapted from the self-reflection dimension of Yan’s (2018) SaPS and revised for AI interaction contexts. It measured students’ critical processing of AI-generated content. A sample item was “I compare and verify AI-generated answers against my own understanding.” Cronbach’s alpha was 0.82. The single-factor CFA indices were χ^2^/df = 1.660, CFI = 0.998, TLI = 0.994, RMSEA = 0.036, and SRMR = 0.012.

#### 3.2.9. Applying AI Output (AA)

The applying AI output scale included four items adapted from the self-adjustment dimension of Yan’s (2018) SaPS and informed by the feedback use framework of (Hattie & Timperley, 2007). It measured students’ self-reported use of AI-generated information in learning activities. A sample item was “I use AI-generated suggestions to improve my work.” Cronbach’s alpha was 0.85. The single-factor CFA indices were χ^2^/df = 1.693, CFI = 0.998, TLI = 0.995, RMSEA = 0.037, and SRMR = 0.011.

### 3.3. Data Analysis

Descriptive statistics were first computed in SPSS 26.0 to summarize sample characteristics and the means and standard deviations of all study variables. Because all valid questionnaires were fully completed, there were no item-level missing data in the analytic dataset. Skewness and kurtosis were also examined as a preliminary check of whether the data were suitable for structural equation modeling.

The main analyses were conducted in Mplus 8.3. First, confirmatory factor analysis (CFA) was performed to evaluate the nine-factor measurement model. Standardized factor loadings, composite reliability (CR), and average variance extracted (AVE) were examined to assess internal consistency and convergent validity. Discriminant validity was evaluated by comparing the square roots of AVE with inter-construct correlations (Fornell & Larcker, 1981a). After the adequacy of the measurement model had been established, correlations among the study variables were examined. Because all variables were collected through self-report questionnaires at a single time point, a single-factor CFA model was also compared with the hypothesized measurement model to assess the potential influence of common method bias (Podsakoff et al., 2003). A full structural equation model (SEM) was then estimated to test the hypothesized paths. Seeking AI help, evaluating AI responses, and applying AI output were specified as three related but distinct outcome variables, and their residuals were allowed to correlate. Model fit was evaluated using chi-square/df, CFI, TLI, RMSEA, and SRMR. Following (Hu & Bentler, 1999), values of χ^2^/df < 3, CFI and TLI close to or above 0.95, RMSEA below 0.06, and SRMR below 0.08 were taken to indicate good model fit. Finally, indirect effects were tested using 5000 bias-corrected bootstrap samples, and significance was determined by whether the 95% confidence interval excluded zero (Hayes & Scharkow, 2013).

## 4. Results

### 4.1. Common Method Bias Test

Because all variables were collected through self-report at a single time point, common method bias was examined first. Specifically, a single-factor CFA model in which all 39 items loaded on one latent factor was compared with the hypothesized nine-factor measurement model. The single-factor model showed poor fit, chi-square = 5441.20, df = 702, *p* < 0.001, χ^2^/df = 7.75, CFI = 0.574, TLI = 0.551, RMSEA = 0.115, and SRMR = 0.101, whereas the nine-factor model showed good fit, chi-square = 1256.85, df = 666, *p* < 0.001, χ^2^/df = 1.89, CFI = 0.947, TLI = 0.941, RMSEA = 0.042, and SRMR = 0.052. The difference in CFI between the two models was 0.373, which was substantially larger than the conventional benchmark of 0.10 (Cheung & Rensvold, 2002). Together with the procedural remedies used in the study, these results suggest that common method bias was not a major threat in the present dataset.

### 4.2. Descriptive Statistics and Correlations

Table 2 presents the means, standard deviations, intercorrelations, and square roots of AVE values for all variables. Skewness values ranged from −0.42 to 0.05, and kurtosis values ranged from −0.63 to −0.24, indicating that the data were suitable for SEM analysis. Among the antecedent variables, instrumental attitude (IAT) had the highest mean (M = 3.71, SD = 0.83), whereas AI anxiety (AIA) had the lowest mean (M = 2.92, SD = 0.91). Among the three behavioral dimensions, evaluating AI responses (EA) had the highest mean (M = 3.61, SD = 0.83), whereas applying AI output (AA) had the lowest mean (M = 3.39, SD = 0.93).

The correlation results were generally consistent with theoretical expectations. Affective attitude, instrumental attitude, subjective norms, perceived behavioral control, and intention were all significantly and positively correlated, with coefficients ranging from 0.34 to 0.61. AI anxiety was significantly and negatively correlated with all other variables, with coefficients ranging from −0.14 to −0.36. The correlations among the three behavioral dimensions ranged from 0.43 to 0.47, suggesting that they were related but not redundant.

### 4.3. Measurement Model

The nine-factor CFA model demonstrated good fit to the data, χ^2^ = 1256.85, *p* < 0.001, χ^2^/df = 1.89, CFI = 0.947, TLI = 0.941, RMSEA = 0.042, and SRMR = 0.052, indicating that the measurement model was acceptable overall. As reported in Appendix A, CR values ranged from 0.813 to 0.895, all above the recommended cutoff of 0.70, and AVE values ranged from 0.523 to 0.668, all above the recommended cutoff of 0.50 (Fornell & Larcker, 1981b). Standardized factor loadings ranged from 0.642 to 0.863, suggesting that the items adequately represented their respective constructs. Cronbach’s alpha coefficients, reported in the measure descriptions, ranged from 0.81 to 0.90, further supporting the internal consistency of the measures. Detailed item-level factor loadings, SMC values, and construct-level reliability and validity indices are reported in Appendix A.

### 4.4. SEM Analysis

After the adequacy of the measurement model had been established, the extended TPB structural model was estimated. The structural model showed good fit, χ^2^ (675) = 1275.14, *p* < 0.001, χ^2^/df = 1.89, CFI = 0.996, TLI = 0.995, RMSEA = 0.042, and SRMR = 0.052. Detailed fit indices for the research model are presented in Appendix A.

With respect to Research Question 1, the results showed that affective attitude (β = 0.182, *p* < 0.001), instrumental attitude (β = 0.447, *p* < 0.001), subjective norms (β = 0.374, *p* < 0.001), and perceived behavioral control (β = 0.237, *p* < 0.001) all significantly and positively predicted intention. Thus, H1a, H1b, H1c, and H1d were supported. AI anxiety did not significantly predict intention (β = 0.056, *p* = 0.123), and H1e was not supported. The model explained 57.3% of the variance in intention (R^2^ = 0.573).

With respect to Research Question 2, intention significantly and positively predicted seeking AI help (β = 0.325, *p* < 0.001), evaluating AI responses (β = 0.413, *p* < 0.001), and applying AI output (β = 0.440, *p* < 0.001), supporting H2a. Perceived behavioral control also significantly and positively predicted seeking AI help (β = 0.369, *p* < 0.001), evaluating AI responses (β = 0.262, *p* < 0.001), and applying AI output (β = 0.283, *p* < 0.001), supporting H2b. At the same time, AI anxiety significantly and negatively predicted all three behavioral dimensions: seeking AI help (β = −0.112, *p* = 0.004), evaluating AI responses (β = −0.131, *p* < 0.001), and applying AI output (β = −0.247, *p* < 0.001), supporting H2c. The variance explained in the three behavioral outcomes was 43.0% for seeking AI help and 38.5% for both evaluating AI responses and applying AI output. Table 3 presents the standardized path coefficients and hypothesis testing results for all hypothesized paths.

### 4.5. Indirect Effects

To further examine whether the antecedent variables were indirectly related to the three behavioral dimensions through intention, bias-corrected bootstrap estimation with 5000 resamples was conducted. As shown in Table 4, the four TPB core predictors—namely, affective attitude, instrumental attitude, subjective norms, and perceived behavioral control—all showed significant indirect effects on the three behavioral dimensions through intention, with standardized indirect effects ranging from 0.047 to 0.142.

By contrast, the indirect effects of AI anxiety on the three behavioral dimensions through intention were all nonsignificant, and the 95% confidence intervals for these effects all included zero (Hayes & Scharkow, 2013). This result is consistent with the structural path analysis reported above: AI anxiety did not significantly predict intention, and its associations with the behavioral dimensions were therefore reflected primarily in direct rather than indirect pathways.

## 5. Discussion

This study used an extended TPB framework to examine high school students’ intentions and behaviors regarding AI learning tool use. Overall, the findings offered strong support for the proposed model and revealed a theoretically meaningful pattern. The core TPB variables were consistently associated with intention and behavior, whereas AI anxiety showed no significant association with intention but exerted significant negative effects on all three behavioral dimensions. This pattern suggests that cognitive evaluations and perceived social-control conditions remain central to intention formation, while AI-specific emotional concerns become more consequential at the stage of behavioral enactment. The major findings are discussed below.

### 5.1. Predictors of Intention

First, affective attitude, instrumental attitude, subjective norms, and perceived behavioral control all significantly and positively predicted intention to use AI learning tools. This result is fully consistent with the core propositions of TPB and with prior studies on educational technology use and learning-related behavior (Armitage & Conner, 2001; Chai et al., 2020; Yan et al., 2020a). Notably, instrumental attitude emerged as the strongest predictor of intention (β = 0.447), followed by subjective norms (β = 0.374), perceived behavioral control (β = 0.237), and affective attitude (β = 0.182). The ordering of these effects is informative because it suggests that high school students’ willingness to use AI learning tools is shaped more strongly by utilitarian judgments and normative considerations than by the enjoyment of using the tools.

The prominence of instrumental attitude indicates that students evaluate AI learning tools primarily in terms of whether the tools can improve efficiency, clarify misunderstandings, and support academic performance. This finding echoes the broader technology acceptance literature, in which perceived usefulness often outweighs affective appeal in predicting intention (Scherer & Teo, 2019). In the present context, such a pattern is especially plausible because high school learning is highly goal-directed and tightly organized around assignments, examinations, and time pressure. Students are therefore likely to approach AI tools less as intrinsically interesting technologies and more as potential means for solving concrete learning problems.

Subjective norms also showed a comparatively strong effect, underscoring the importance of the social environment in shaping adolescents’ technology-related intentions (Teo et al., 2016). High school students typically operate in settings characterized by close teacher supervision, parental oversight, and salient peer influence. Under such conditions, whether important others view AI use as legitimate, useful, or risky is likely to affect not only students’ perceived social approval but also their readiness to translate favorable evaluations into intention. The present findings therefore reinforce the view that social expectations remain particularly consequential in secondary education settings.

Perceived behavioral control significantly predicted intention as well, indicating that students are more willing to use AI learning tools when they believe they possess sufficient knowledge, operational skills, and opportunity to do so effectively. In the AI context, perceived control is likely to involve more than technical access alone. It also includes confidence in asking effective questions, interpreting responses, and managing possible inaccuracies or misuse. This helps to explain why perceived behavioral control contributes to intention formation in addition to its later role in predicting behavior.

### 5.2. Predictors of Behavior

The study further showed that intention and perceived behavioral control significantly predicted all three behavioral dimensions: seeking AI help, evaluating AI responses, and applying AI output. This result supports the TPB proposition that intention is a proximal determinant of behavior (Ajzen, 2020) and further indicates that students’ stated willingness to use AI is meaningfully translated into concrete engagement with AI-supported learning activities.

At the same time, the effects were not uniform across the three outcomes. Intention showed the strongest association with applying AI output (β = 0.440), followed by evaluating AI responses (β = 0.413) and seeking AI help (β = 0.325). This gradient suggests that the more consequential the behavior, the more closely it is tied to deliberate intention. Asking AI for help is a relatively low-threshold action that can be initiated quickly and reversed easily. Evaluating AI responses requires more cognitive investment, but the student remains at the stage of appraisal. Applying AI output, by contrast, involves integrating externally generated content into one’s own learning products or strategies, thereby increasing the salience of accuracy, originality, and responsibility. It is therefore reasonable that this dimension is most strongly linked to intention.

Perceived behavioral control showed a different pattern, with the strongest effect on seeking AI help (β = 0.369), followed by applying AI output (β = 0.283) and evaluating AI responses (β = 0.262). This finding suggests that students’ sense of capability is particularly important at the entry point of AI use. Initiating interaction with AI requires basic operational competence, confidence in formulating prompts, and the practical ability to access and use the tool. By comparison, evaluating and applying AI output depend not only on capability but also on standards of judgment, task demands, and academic norms. The somewhat weaker coefficients for these later stages may therefore reflect the fact that behavioral control alone is insufficient to explain them.

Taken together, these results support the decision to conceptualize AI learning tool use as multidimensional rather than as a single undifferentiated outcome. Different predictors mattered across seeking, evaluating, and applying, and the pattern of coefficients suggests that these behaviors vary in threshold, complexity, and perceived consequence. A multidimensional perspective therefore captures important internal differences in how students engage with AI during the learning process.

### 5.3. The Differential Role of AI Anxiety

The most distinctive finding of the study is that AI anxiety did not significantly predict intention (β = 0.056, *p* = 0.123), yet it significantly and negatively predicted seeking AI help (β = −0.112), evaluating AI responses (β = −0.131), and especially applying AI output (β = −0.247). This pattern suggests that anxiety toward AI does not substantially reshape students’ stated willingness to use AI learning tools, but it does constrain what they actually do with those tools. Put differently, AI anxiety appears to operate less as a determinant of intention formation and more as an inhibitor of behavioral enactment.

This result is consistent with prior technology adoption research showing that anxiety-related variables are often more closely tied to behavioral barriers than to deliberative intention judgments (Venkatesh, 2000). It also aligns with evidence that extended variables in technology adoption models may affect downstream behavior even when their effects on intention are weak or nonsignificant. The present study extends this line of reasoning to AI-supported learning in secondary education and indicates that AI-specific affect deserves separate theoretical attention rather than being treated as interchangeable with the classical TPB predictors.

One plausible interpretation is that, when reporting intention, students rely primarily on broad judgments about usefulness, social expectations, and personal capability. Such judgments are relatively cognitive and reflective. By contrast, anxiety may become more influential in actual moments of use, when students must decide whether to trust AI-generated information, whether relying on AI is appropriate, and whether using AI output could expose them to errors or academic risk. Under these conditions, emotional concern may suppress action even when general willingness remains intact. This interpretation is also consistent with the nonsignificant indirect effects of AI anxiety through intention, which indicate that its influence was expressed primarily through direct pathways to behavior.

The strongest negative association was found for applying AI output. This result is theoretically important because application is the stage at which students move from consultation to incorporation. Compared with asking questions or evaluating responses, adopting AI-generated content in one’s own learning products requires a higher degree of responsibility and commitment. Students may hesitate because they are uncertain about the reliability of the content, concerned about becoming overdependent, or worried that such use could violate academic expectations. The relatively large coefficient for this path therefore suggests that AI anxiety is especially consequential when AI use approaches actual academic adoption rather than preliminary exploration.

## 6. Conclusions, Limitations, and Implications

### 6.1. Conclusions

This study investigated high school students’ intentions and behaviors in using AI learning tools through an extended TPB framework. The findings show that intention is primarily shaped by instrumental attitude, subjective norms, perceived behavioral control and, to a lesser extent, affective attitude. In turn, intention and perceived behavioral control significantly predict seeking AI help, evaluating AI responses, and applying AI output. By contrast, AI anxiety does not significantly influence intention but directly inhibits all three behavioral dimensions, especially the application of AI output. Taken together, these results indicate that high school students’ AI learning tool use is jointly structured by cognitive evaluations, perceived social and behavioral conditions, and AI-specific emotional barriers. The extended TPB model therefore offers a useful framework for explaining adolescent engagement with AI-supported learning.

### 6.2. Theoretical and Practical Implications

This study has several theoretical implications. First, it extends the TPB framework by incorporating AI anxiety and demonstrates that AI-related affect does not simply replicate the function of the classical TPB variables. Whereas attitude, subjective norms, and perceived behavioral control primarily shape intention, AI anxiety appears to bypass intention and operate directly at the behavioral level. This finding suggests that future TPB-based research on AI-supported learning should distinguish more carefully between reflective cognitive appraisals and domain-specific emotional inhibition. Second, the study conceptualizes AI learning tool use as a multidimensional construct comprising seeking, evaluating, and applying. Because the predictors showed different effect sizes across these dimensions, the findings indicate that treating AI use as a single global outcome may obscure meaningful variation in how learners interact with AI across the learning process.

The practical implications are equally clear. If schools aim to promote responsible and effective AI-assisted learning, they should not focus only on access to tools. They also need to strengthen students’ instrumental understanding of AI’s learning value, enhance perceived behavioral control through AI literacy instruction and guided practice, and establish more consistent normative expectations across teachers, parents, and peers. At the same time, schools should address students’ AI-related concerns explicitly, especially those involving misinformation, overreliance, and academic integrity. The present findings suggest that reducing such concerns is important not merely for improving attitudes, but for supporting actual behavioral engagement with AI in everyday learning.

### 6.3. Limitations and Future Research Directions

Several limitations should be acknowledged. First, the cross-sectional design does not support causal inference (Maxwell & Cole, 2007). Although the hypothesized paths were derived from TPB theory, the data were collected at a single time point, and the observed associations may still reflect reciprocal influence or omitted variables. Longitudinal, panel, or experience sampling designs would be better suited to examining how changes in attitudes, anxiety, and perceived control translate into later behavioral change.

Second, all constructs were measured through student self-reports, which leaves the data vulnerable to social desirability, response tendencies, and retrospective bias, and also constrains the assessment of common method bias. Several procedural remedies were implemented during instrument design and administration, and a single-factor confirmatory factor analysis was conducted as an ex post statistical screen. As noted in Section 4.1, the single-factor contrast is nevertheless a limited diagnostic, because method variance can be multidimensional and need not load on a single global factor. The present results should therefore be interpreted with the residual possibility of method effects in mind. Future research should adopt more sensitive procedures for assessing common method bias, such as the inclusion of a pre-specified marker variable or a common-latent-factor model at the design stage, and complement self-report measures with more objective behavioral indicators from AI platforms, such as query logs, usage duration, revision traces, or rates of accepting AI suggestions.

Third, the model focused on predictors of AI learning tool use but did not include downstream learning outcomes. As a result, it remains unclear whether seeking, evaluating, and applying AI output contribute differently to academic performance, conceptual understanding, or self-regulated learning. Connecting the present behavioral framework to measurable learning outcomes would substantially increase its practical and theoretical value. Similarly, intention was measured as a unitary construct whereas behavior was modeled in three dimensions. Future research could develop dimension-specific intention measures and examine whether a fully matched intention–behavior model offers greater predictive precision.

Finally, the sample was drawn from three urban public high schools in Hangzhou where AI tools had already been introduced into educational practice. While this sampling strategy improved the relevance of participants’ responses, it also limits the generalizability of the findings. Students in rural regions, private schools, or contexts with less institutional support for AI integration may show different patterns. Cross-regional, cross-school-type, and cross-cultural replication is therefore needed to test the robustness and boundary conditions of the present results. Within these boundary conditions, one further interpretive caveat concerns H2c: although the hypothesis was theoretically motivated, the relative magnitude of the direct versus the intention-mediated effects of AI anxiety was treated as an open empirical question rather than as a precise prior prediction. The pattern observed in the present data, in which the direct effects on the three behavioral dimensions were significant whereas the indirect effects through intention were not, is consistent with the dual-route reasoning outlined in Section 2.4, but it should be replicated in independent samples before strong inferences about the relative dominance of the two routes can be drawn.

## Figures and Tables

**Table 1:** Descriptive Statistics of the Sample (*N* = 513).

Variable	Category	Frequency	Percentage (%)
Gender	Male	262	51.1
	Female	251	48.9
Grade	10	260	50.7
	11	253	49.3

**Table 2:** Descriptive Statistics, Correlations and AVE Square Roots (*N* = 513).

Factor	M	SD	AAT	IAT	SNS	PBC	AIA	INT	SA	EA	AA
AAT	3.56	0.92	**0.817**								
IAT	3.71	0.83	0.42 **	**0.764**							
SNS	3.4	0.87	0.39 **	0.34 **	**0.723**						
PBC	3.56	0.84	0.47 **	0.37 **	0.40 **	**0.767**					
AIA	2.92	0.91	−0.22 **	−0.24 **	−0.14 **	−0.30 **	**0.762**				
INT	3.43	0.91	0.51 **	0.61 **	0.56 **	0.52 **	−0.19 **	**0.757**			
SA	3.55	0.88	0.39 **	0.37 **	0.42 **	0.57 **	−0.30 **	0.55 **	**0.784**		
EA	3.61	0.83	0.41 **	0.46 **	0.42 **	0.52 **	−0.30 **	0.53 **	0.46 **	**0.733**	
AA	3.39	0.93	0.36 **	0.40 **	0.40 **	0.51 **	−0.36 **	0.51 **	0.47 **	0.43 **	**0.764**

Note. *N* = 513. ** *p* < 0.01. AAT = affective attitude; IAT = instrumental attitude; SNS = subjective norms; PBC = perceived behavioral control; AIA = AI anxiety; INT = intention; SA = seeking AI help; EA = evaluating AI responses; AA = applying AI output. Bold values on the diagonal are the square roots of AVE.

**Table 3:** Standardized Path Coefficients and Hypothesis Testing Results.

DV	IV	Std.Est.	S.E.	z	*p*	R^2^	Hypothesis
INT	AAT	0.182	0.044	4.120	<0.001	0.573	Supported H1a
	IAT	0.447	0.047	9.500	<0.001		Supported H1b
	SNS	0.374	0.050	7.490	<0.001		Supported H1c
	PBC	0.237	0.048	4.900	<0.001		Supported H1d
	AIA	0.056	0.036	1.540	0.123		Not supported H1e
SA	INT	0.325	0.048	6.730	<0.001	0.43	Supported H2a-SA
	PBC	0.369	0.057	6.500	<0.001		Supported H2b-SA
	AIA	−0.112	0.039	−2.920	0.004		Supported H2c-SA
EA	INT	0.413	0.050	8.250	<0.001	0.385	Supported H2a-EA
	PBC	0.262	0.056	4.710	<0.001		Supported H2b-EA
	AIA	−0.131	0.039	−3.390	<0.001		Supported H2c-EA
AA	INT	0.440	0.058	7.590	<0.001	0.385	Supported H2a-AA
	PBC	0.283	0.066	4.300	<0.001		Supported H2b-AA
	AIA	−0.247	0.047	−5.260	<0.001		Supported H2c-AA

Note. AAT = affective attitude; IAT = instrumental attitude; SNS = subjective norms; PBC = perceived behavioral control; AIA = AI anxiety; INT = intention; SA = seeking AI help; EA = evaluating AI responses; AA = applying AI output.

**Table 4:** Indirect Effects on AI Learning Tool Use Behaviors.

Indirect Effect	β	SE	z	*p*	95% CI Lower	95% CI Upper
Path to Seeking AI Help						
AAT → INT → SA	0.05	0.013	3.91	<0.001	0.026	0.076
IAT → INT → SA	0.138	0.019	7.2	<0.001	0.104	0.178
SNS → INT → SA	0.105	0.017	6.34	<0.001	0.074	0.138
PBC → INT → SA	0.074	0.014	5.1	<0.001	0.047	0.104
AIA → INT → SA	0.013	0.011	1.27	0.202	−0.007	0.035
Path to Evaluating AI Responses						
AAT → INT → EA	0.047	0.012	3.98	<0.001	0.025	0.072
IAT → INT → EA	0.132	0.02	6.71	<0.001	0.095	0.172
SNS → INT → EA	0.1	0.016	6.4	<0.001	0.071	0.132
PBC → INT → EA	0.071	0.016	4.48	<0.001	0.043	0.104
AIA → INT → EA	0.013	0.01	1.26	0.207	−0.007	0.034
Path to Applying AI Output						
AAT → INT → AA	0.051	0.013	3.87	<0.001	0.027	0.078
IAT → INT → AA	0.142	0.021	6.74	<0.001	0.101	0.185
SNS → INT → AA	0.108	0.018	5.95	<0.001	0.074	0.145
PBC → INT → AA	0.076	0.016	4.61	<0.001	0.046	0.111
AIA → INT → AA	0.014	0.011	1.26	0.207	−0.007	0.037

Note. β = standardized estimate. CI = 95% confidence interval based on 5000 bias-corrected bootstrap resamples.

## Data Availability

The datasets generated during and analyzed during the current study are available from the corresponding author on reasonable request.

## Supplementary Materials

The following supporting information can be downloaded at: https://www.mdpi.com/article/10.3390/bs16050736/s1, Table S1: Survey questionnaire used in the present study.

## Appendix A. Measurement Model Details

**Table A1:** Measurement Model: Factor Loadings, Reliability, and Validity Indices.

Factors	Items	Std.	SMC	CR	AVE
AAT	AAT1	0.855	0.731	0.889	0.668
	AAT2	0.797	0.634		
	AAT3	0.863	0.745		
	AAT4	0.748	0.559		
IAT	IAT1	0.856	0.733	0.848	0.584
	IAT2	0.74	0.547		
	IAT3	0.718	0.515		
	IAT4	0.735	0.541		
SNS	SNS1	0.777	0.604	0.813	0.523
	SNS2	0.652	0.426		
	SNS3	0.786	0.618		
	SNS4	0.665	0.443		
PBC	PBC1	0.794	0.63	0.895	0.588
	PBC2	0.748	0.559		
	PBC3	0.837	0.701		
	PBC4	0.683	0.466		
	PBC5	0.796	0.634		
	PBC6	0.732	0.536		
AIA	AIA1	0.802	0.644	0.873	0.58
	AIA2	0.728	0.53		
	AIA3	0.705	0.497		
	AIA4	0.779	0.607		
	AIA5	0.789	0.623		
INT	INT1	0.857	0.735	0.841	0.573
	INT2	0.648	0.42		
	INT3	0.823	0.677		
	INT4	0.679	0.461		
SA	SA1	0.782	0.611	0.864	0.615
	SA2	0.785	0.617		
	SA3	0.768	0.59		
	SA4	0.8	0.64		
EA	EA1	0.751	0.563	0.822	0.537
	EA2	0.719	0.517		
	EA3	0.809	0.654		
	EA4	0.642	0.412		
AA	AA1	0.817	0.667	0.848	0.584
	AA2	0.721	0.52		
	AA3	0.825	0.68		
	AA4	0.683	0.467		

Note. Std. = standardized factor loading; SMC = squared multiple correlation; CR = composite reliability; AVE = average variance extracted. AAT = affective attitude; IAT = instrumental attitude; SNS = subjective norms; PBC = perceived behavioral control; AIA = AI anxiety; INT = intention; SA = seeking AI help; EA = evaluating AI responses; AA = applying AI output.

**Table A2:** Fit Indices.

Fit Indices	Criteria	Research Model	Result
χ^2^	-	1274.14	-
df	-	675	-
χ^2^/df	1 < χ^2^/df < 3	1.89	Acceptable
CFI	≥0.95	0.996	Acceptable
TLI	≥0.95	0.995	Acceptable
RMSEA	<0.06	0.042	Acceptable
SRMR	≤0.08	0.052	Acceptable
