# Supplementary material for: Predictors of High School Students’ Intentions and Behaviors in Using AI Learning Tools: An Extended Theory of Planned Behavior Approach
Source: Behav Sci (Basel). 2026 May 9;16(5):736. doi: 10.3390/bs16050736 (PMC13203645; doi:10.3390/bs16050736)
Supplement: Supplementary file 1 [file behavsci-16-00736-s001.zip › behavsci-4253955-supplementary.pdf]

Survey Questionnaire

Demographic questions

What is your gender?

- Male ☐
- Female ☐

What is your grade level?

- Grade 10 ☐
- Grade 11 ☐

Scale section

Please select the rating that best describes your experience, where 1 means "Strongly disagree," 2 means "Disagree," 3 means "Neutral," 4 means "Agree," and 5 means "Strongly agree."

Table S1. Survey Questionnaire used in the present study.

**Variable**	**Items**
Affective attitude	
AAT1	Using AI learning tools is enjoyable.
AAT2	Using AI learning tools is interesting to me.
AAT3	Using AI learning tools makes me feel satisfied.
AAT4	Using AI learning tools is a positive experience.
Instrumental attitude	
IAT1	Using AI learning tools helps me understand my strengths and weaknesses in learning.
IAT2	Using AI learning tools helps improve my learning efficiency.
IAT3	Using AI learning tools is valuable for my academic work.
IAT4	Using AI learning tools makes learning easier.
Subjective norms	
SNS1	My teachers think I should use AI learning tools to support my learning.
SNS2	My parents/guardians support my use of AI learning tools.
SNS3	My classmates think using AI learning tools is a good thing.
SNS4	Most people who are important to me think I should use AI learning tools.
Perceived behavioral control	
PBC1	I have sufficient knowledge to use AI learning tools effectively.
PBC2	I am confident that I can use AI learning tools correctly.
PBC3	Whether to use AI learning tools is entirely up to me.
PBC4	I have enough resources and opportunities to use AI learning tools.
PBC5	Using AI learning tools is easy for me.
PBC6	I can independently choose when and how to use AI learning tools.
AI anxiety	
AIA1	I feel anxious when I need to use AI tools for learning tasks.
AIA2	I worry that I cannot correctly understand the answers given by AI tools.
AIA3	Thinking about the application of AI technology in learning makes me uneasy.
AIA4	I worry that over-reliance on AI tools will affect my ability to think independently.
AIA5	When using AI learning tools, I worry about my academic integrity being questioned.
Intention	
INT1	I plan to use AI learning tools to support my learning in the future.
INT2	I am willing to try using AI learning tools to assist my studies.

INT3	I intend to continue using AI learning tools in my daily learning.
INT4	If conditions permit, I would frequently use AI learning tools.
Seeking AI help	
SA1	When I have learning questions, I seek help from AI tools.
SA2	I proactively use AI tools to get explanations and guidance for learning.
SA3	When I do not understand a concept, I ask AI tools for help.
SA4	I use AI tools to find quality learning resources and examples.
Evaluating AI responses	
EA1	I carefully consider AI-generated responses before deciding whether to use them.
EA2	I compare and verify AI-generated answers against my own understanding.
EA3	I critically evaluate the accuracy and reasonableness of AI-generated content.
EA4	I judge whether AI-generated content is applicable to my current learning needs.
Applying AI output	
AA1	I use AI-generated suggestions to improve my work.
AA2	I adjust my learning strategies based on feedback provided by AI.
AA3	I apply AI-generated explanations to solve actual learning problems.
AA4	I strengthen my learning in targeted areas based on weaknesses identified by AI.
